# LDC000067, a CDK9 inhibitor, unveils promising results in suppressing influenza virus infections *in vitro* and *in vivo*

**DOI:** 10.1128/aac.01172-24

**Published:** 2024-11-20

**Authors:** Mingxin Zhang, Xiaoqin Lian, Yarou Gao, Lefang Jiang, Zhuogang Li, Haonan Zhang, Yue Su, Qun Peng, Xulin Chen

**Affiliations:** 1 Department of Immunology and Microbiology, Institute of Medical Microbiology, College of Life Science and Technology, Jinan University 47885 , Guangzhou, Guangdong, China; IrsiCaixa Institut de Recerca de la Sida, Barcelona, Spain

**Keywords:** LDC000067, CDK9, antiviral target, RNA transcription, nuclear import of vRNPs

## Abstract

Influenza virus infections continue to pose a significant threat to public health. Current anti-influenza drugs target viral proteins; however, the emergence of resistant strains has hampered their effectiveness. Fortunately, as with most viruses, influenza virus depends on various host factors during its replication cycle and in pathogenicity. Therefore, the manipulation of key host factors for viral replication to combat influenza has garnered increased attention due to its lesser tendency to induce viral mutation. Cyclin-dependent kinases (CDKs) are a family of protein kinases that regulate various cellular processes, including the cell cycle and transcription. While the specific involvement of CDKs in the transcription of influenza virus genes is less extensively studied than their roles in the cell cycle, some evidence suggests their potential contributions as anti-influenza drugs. Here, we report that LDC000067 (LDC), a highly specific CDK9 inhibitor, not only strongly suppressed influenza virus replication *in vitro* and *in vivo* but also emerged as a potential candidate for anti-influenza virus agents. Further investigation revealed that inhibition of CDK9 by LDC treatment and CDK9 silencing disrupts viral RNA transcription and the nuclear import of vRNPs, significantly suppressing influenza virus replication. Mechanistically, we showed that LDC treatment and CDK9 silencing reduce Pol II expressions, a requisite host protein for viral RNA transcription. Altogether, our findings indicate that CDK9 could be a promising target for developing antivirals against influenza virus infections, and LDC, with its strong anti-influenza properties, instills confidence in its potential as an effective anti-influenza agent.

## INTRODUCTION

Influenza virus (IFV) is a respiratory pathogen responsible for seasonal epidemics. It can also cause major influenza pandemics and pose a global health threat (1). The annual global estimate for deaths due to respiratory complications from influenza is approximately 650,000 (2). Vaccination against influenza is considered the first line to prevent influenza transmission; however, its effectiveness varies depending on the circulating virus strain, and the provided protection for the elderly and young children is more limited (3). Therefore, alternative strategies are required for efficiently controlling influenza outbreaks. Neuraminidase inhibitors (such as oseltamivir and zanamivir) and viral RNA polymerase inhibitors (such as baloxavir marboxil) are essential in reducing the risk of complications and death from influenza. However, the effectiveness of these antiviral drugs is limited by the rapid emergence of drug-resistant IFVs (4). Consequently, there is an urgent need to develop novel anti-IFV agents with less tendency to induce viral mutation.

Belonging to the Orthomyxoviridae family, IFV has a genome of eight segmented negative-sense RNA (5, 6). IFV viral RNA, along with its heterotrimeric RNA-dependent RNA polymerases (RdRp, PB1, PB2, and PA) and nucleocapsid protein (NP), forms replication and transcriptionally active ribonucleoprotein complexes (vRNPs) (7, 8). Host cell infection by IFV starts with its hemagglutinin (HA) binding to the host cell’s sialic acid residues. Receptor-mediated endocytosis helps the virus to enter the host cell’s endosome. The endosome’s low pH induces HA conformational change resulting in viral and endosomal membrane fusion (9, 10), and subsequent vRNPs release into the host cell cytoplasm (11–13). The nuclear localization signals (NLSs) in NP facilitate the nuclear import of vRNPs through the cellular importin-α/β-dependent nuclear import pathway (14–17). Once entering the nucleus, the heterotrimeric viral RdRp mediates the transcription of viral mRNAs and replication of vRNAs (18). Following viral replication in the nucleus, newly synthesized vRNPs are exported from the nucleus to the cytoplasm through a CRM1-dependent pathway, reaching the viral assembly sites by the plasma membrane. The mature virions are ultimately released via extracellular budding (19, 20).

Like all viruses, IFV hijacks the host cell’s biological processes for their own replication (21). The interaction between host and virus is pivotal in regulating viral replication and pathogenicity (22). Therefore, host factors involved in the IFV life cycle could potentially be targeted to interfere with virus replication (23). Recently, a growing number of viral and host factors associated with IFV infection have been reported (24–27). Identifying key host factors and developing antiviral agents targeting these factors are of great importance in developing novel antiviral therapies and mitigating viral drug resistance. Out of all the host factors, cellular kinases play a variety of important roles during multiple stages of the virus’s life cycle (28, 29). To date, more than 28 host kinases are implicated in regulating IFV replication by different mechanisms (30). The cyclin-dependent kinases (CDKs), a family of serine/threonine kinases, primarily function in regulating the cell cycle by interacting with specific cell cycle regulatory cyclins (31). In recent years, emerging evidence has underscored the involvement of CDKs in viral infections. Multiple viruses, such as human immunodeficiency virus (HIV) and herpes simplex virus (HSV), exploit essential CDKs to create a conducive environment for viral replication, either by disrupting cell cycle progression or overcoming cellular restriction factors (32, 33). Therefore, CDKs could be potential host factors for developing novel anti-IFV agents.

Unlike conventional CDKs, CDK9 does not directly govern cell cycle progression, and its activity is believed to be limited to terminally differentiated cells (34). In mammalian cells, CDK9 and cyclins T1 or T2 comprise a subunit of the positive transcription elongation factor b (P-TEF b) multiprotein complex, which initiates the transcriptional elongation of genes by phosphorylating the carboxyterminal domain (CTD) of RNA polymerase II (Pol II) (35). IFV vRNPs specifically target the Pol II CTD when it is phosphorylated, and the form of Pol II is involved in the capping of nascent transcripts (36). Zhang et al. demonstrated that cyclin T1/CDK9 complex interacted with influenza A virus vRNP and mediated its association with the Ser-2-phosphorylated CTD of Pol II *in vitro* (37). This study reported that CDK9 activity is not associated with IFV replication. However, a recent study reported that five new pyrazolopyrimidines, a class of purine analogs, potently inhibit avian IFV H5N1 replication in MDCK cells by targeting cellular CDK9 (38). These conflicting results indicate that the role of CDK9 in IFV infection remains not well clarified. Additionally, the function of CDK9 in IFV infection *in vivo* has not been previously reported.

Here, we reported that LDC000067 (LDC), a highly specific CDK9 inhibitor, exhibited potent inhibition on replication of different IFV strains in A549 and MDCK cells, and provided remarkable protection for IFA-infected mice. Mechanistically, we demonstrated that LDC reduced CDK9 expression. This reduction suppresses mRNA transcription and the nuclear import of vRNPs in A549 cells. Our findings suggest that CDK9 may be a promising antiviral target, and LDC has great potential as a novel anti-influenza agent.

## RESULTS

### LDC inhibits IFV replication *in vitro*

**Fig 1:**
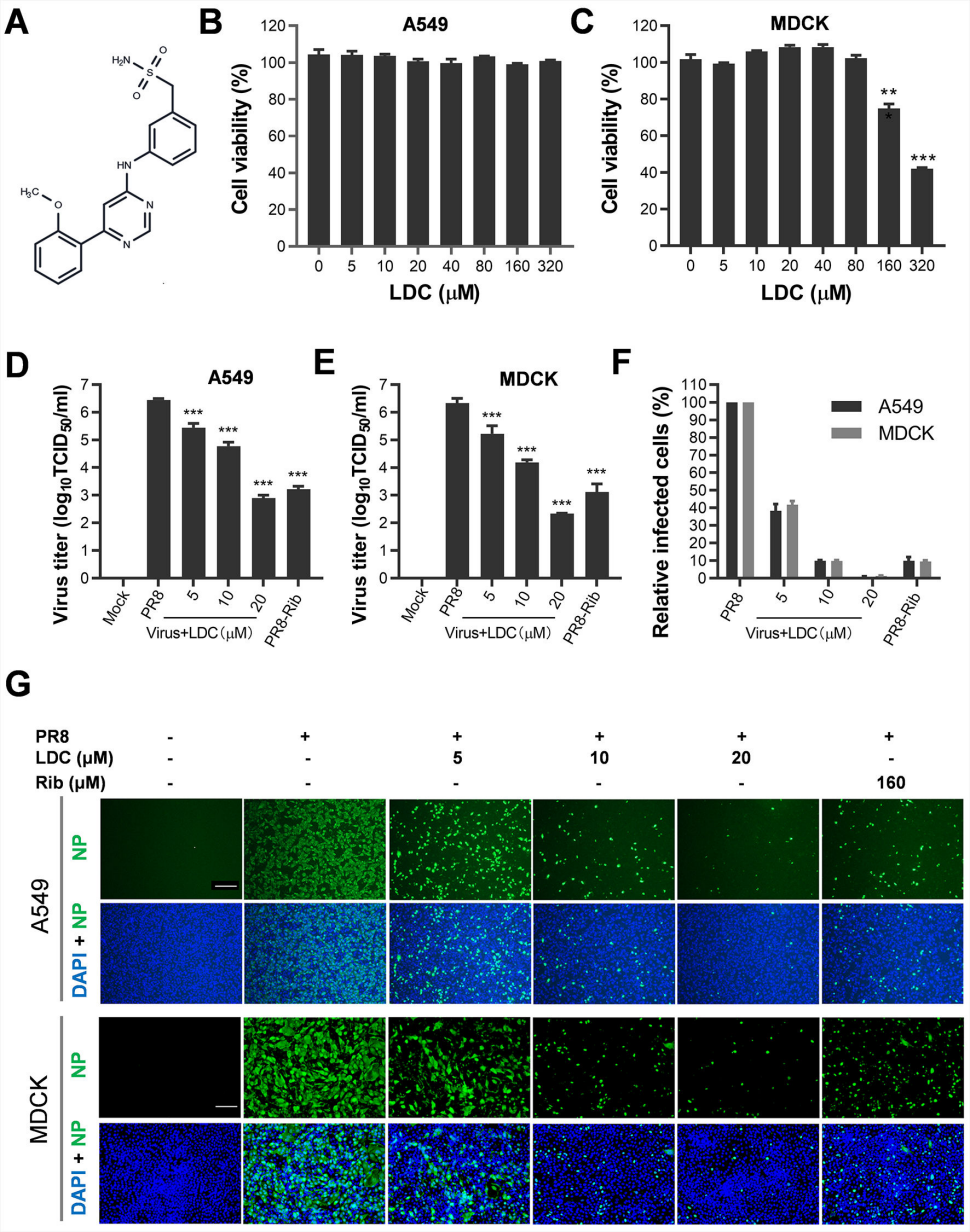
Cytotoxicity and anti-IFV activity of LDC in MDCK and A549 cells. (**A**) Chemical structure of LDC. (**B and C**) LDC cytotoxicity in MDCK and A459 cells was examined using Cell Titer-Glo cell viability assay kits and is presented as relative cell viability of the viable cells in the absence of the compound (set up as 100%) (*n* = 3 independent experiments, data are shown as mean ± SEM). (**D and E**) LDC antiviral activity against IFV (PR8) infection of MDCK and A459 cells was determined by viral titers (*n* = 3 independent experiments, data are shown as mean ± SD). (**F and G**) LDC antiviral activity against IFV infection of A549 and MDCK cells was examined by IFA. Cells grown in 96-well plates were infected with PR8 for 1 h at 37°C and then cultured in fresh media containing various concentrations of LDC. IFA for the viral NP protein was performed at 24 hpi using Alexa Fluor 488-conjugated goat anti-mouse secondary antibody (green). Nuclei were counterstained using 4,6-diamidino-2-phenylindole (DAPI) (blue). Results shown in (F) are the mean values of percentages of PR8-infected cell ratio in LDC-treated groups compared to the dimethyl sulfoxide (DMSO)-treated control (No LDC, set up as 100%) from three independent experiments, and (G) is one representative IFA data set from (F). Scale bar: 200 µm. Statistical significances are denoted by *P < 0.05, **P < 0.01, and ***P < 0.001.

Using a Cell Titer-Glo cell viability assay, we first assessed the cytotoxicity of LDC on A549 and MDCK cells. Serially diluted LDC was added to the cells, and the cell viabilities were evaluated at 48 h post-treatment. As depicted in Fig. 1B and C, the results indicate that LDC did not adversely affect cell viability in A549 cells at concentrations up to 320 µM (Fig. 1B). In contrast, LDC displayed cytotoxic effects on MDCK cells at concentrations exceeding 80 µM. Nevertheless, even at 160 µM, cell viability in MDCK cells remained above 70% (Fig. 1C). These findings suggest that LDC induces minimal cytotoxicity in both A549 and MDCK cells following short-term treatment.

Subsequently, we assessed LDC’s antiviral effects against the IFV in both A549 and MDCK cells. The cells were exposed to the IFV (PR8) with varying concentrations of LDC and then incubated for 24 h. Subsequently, the virus titers in the supernatants were determined using a viral titer titration assay. Our findings, illustrated in Fig. 1D and E, revealed a substantial dose-dependent inhibition of infectious virion production in both A549 and MDCK cells after LDC treatment. The half maximal effective concentrations (EC_50_) of LDC, calculated based on the virus titers of infected cells, were determined to be 5.16 µM for A549 cells and 8.94 µM for MDCK cells. The calculated selective indexes (SIs) for the two cell lines were higher than 62.0 and 19.8, respectively.

We further assessed the antiviral activity of LDC on IFV replication by evaluating NP levels through immunofluorescence microscopy analysis (IFA) in A549 cells, as indicated in Fig. 1F and G, where LDC treatment exhibited a dose-dependent inhibition of NP expression at 24 h post-infection (hpi). Notably, ribavirin, a nucleotide analog, has been reported to effectively suppress IFV replication *in vitro* and *in vivo* (39). Consistent with these reports, our results demonstrated significant inhibition of IFV replication with ribavirin at 120 µM. These findings elucidate the potent inhibitory effect of LDC on IFV replication, which is cell independent.

### LDC exhibits a broad spectrum of antiviral activity against IFV infections

**Fig 2:**
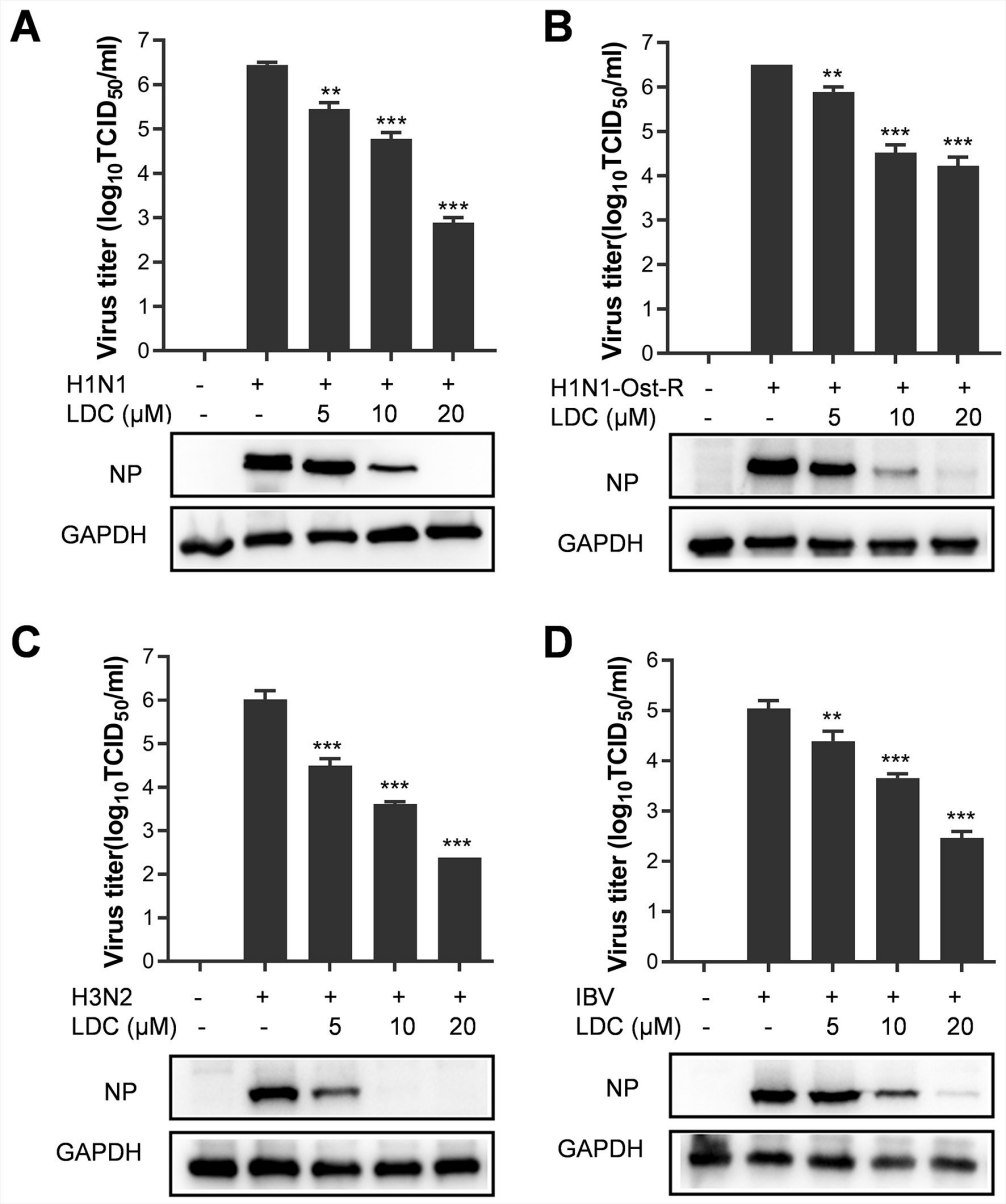
LDC inhibits the replication of various IFV strains in A549 cells. (**A–D**) A549 cells cultured in 24-well plates were infected with multiple strains of IFV (H1N1 pan2009, H1N1 OST-R, H3N2, and IBV) for 1 h at 37°C and then treated with varying concentrations of LDC in fresh media. At 24 hpi, the supernatant and cells were collected and subjected to viral titer titration and Western blot. The presented data represent results from three independent experiments (mean ± SD). Statistical significance was analyzed using one-way analysis of variance (ANOVA). Statistically significant differences are denoted as *P < 0.05, **P < 0.01, and ***P < 0.001.

To further explore the antiviral impact of LDC on diverse subtypes, including an oseltamivir-resistant strain, of IFV replication, A549 cells (5 × 10^5^ cells per well) were cultured in a 12-well plate for 24 h. Subsequently, the cells were infected with IFV strains, including influenza A virus subtypes, A/human/Hubei/1/2009 (H1N1 pan2009), oseltamivir-resistant A/Puerto Rico/8/1934 (H274Y) (H1N1-Ost-R), A/human/Hubei/3/2005 (H3N2), and influenza B virus subtypes B/human/Hubei/1/2007 (IBV) at a multiplicity of infection (MOI) of 0.3. Following a 1-h incubation, the cells were cultured with fresh media containing various concentrations of LDC. Results in Fig. 2 demonstrate the inhibitory effect of LDC on the four distinct IFV strains assessed via viral titers. The EC_50_ values of LDC against the five tested IFV strains on A549 cells ranged from 3.92 to 6.31 µM (Fig. 1 and 2), with corresponding selectivity indices (SIs) exceeding 50.7, as documented in Table 1. These observations indicate that LDC considerably suppressed IFV replication, with this effect being strain independent.

**TABLE 1:** Inhibitory activity of LDC against various IFV strains in A549 cells

Virus strain	PR8	H1N1 pan2009	H1N1 OST-R	H3N2	IBV
EC_50_ (μM)^*a*^	5.16 ± 1.83	3.92 ± 1.15	4.11 ± 0.60	3.96 ± 0.94	6.31 ± 2.35
Selectivity index^*b*^	>62.0	>81.6	>77.9	>80.8	>50.7

^*a*^ EC_50_ denotes the concentration necessary to safeguard 50% of cells from IFV infection by enumerating infected cells through viral titer titration as described in Materials and Methods.

^*b*^ The selectivity index represents the ratio of 50% cell cytotoxic concentration (CC_50_) to EC_50_. The CC_50_ of LDC on A549 exceeds 320 µM. The data are depicted as means ± standard error of results from three independent experiments.

### LDC inhibits IFV replication during the mid-phase of the viral replication cycle

**Fig 3:**
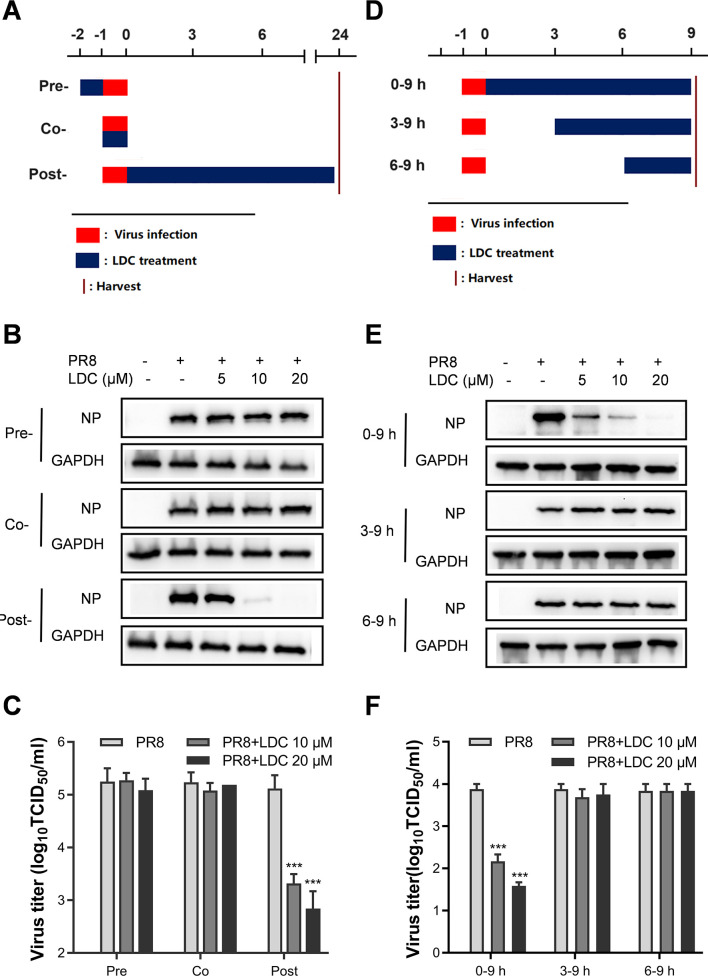
Inhibition on IFV replication is effectively achieved through the early treatment of LDC on A549 cells. (**A–C**) A549 cells were incubated with LDC for 1 h before being infected with PR8 at an MOI of 0.3 for 1 h (Pre-), cells were incubated with PR8 virus and treated with LDC for 1 h (Co-), or cells were incubated with PR8 for 1 h and then cultured in fresh media containing various concentrations of LDC (Post-). The samples were collected at 24 hpi and subjected to Western blot (**B**) and virus titer analysis (**C**). (**D–F**) A549 cells were infected with PR8 (3 MOI) for 1 h. LDC at various concentrations was added to the PR8-infected cells at the indicated time points post-infection, and the viral NP levels and titers were determined at 9 hpi. Statistical significance is indicated by *P < 0.05, **P < 0.01, and ***P < 0.001.

To investigate the impact of LDC on the replication of influenza A virus, we analyzed virus replication, as indicated by NP level and the production of infectious virions, at different time points of LDC addition/removal: pre-treatment (LDC was added 1 h before infection and removed just before infection), co-treatment (simultaneous LDC treatment and virus infection for 1 h), and post-treatment (LDC was added 1 h post-infection and treated for 24 h) (Fig. 3A). The results revealed that post-treatment with LDC demonstrated antiviral activity, resulting in reduced NP expression and virus production, while pre- and co-treatment did not have an impact on IFV NP expression or virus titer (Fig. 3B and C). Subsequent experiments on time-of-addition assay (Fig. 3D) confirmed that immediate addition of LDC after virus infection markedly inhibited viral NP protein expression and virus production. However, adding LDC at 3 h or later post-infection did not decrease NP expression and virus titer (Fig. 3E and F). These results signify that early administration of LDC effectively impedes IFV replication.

**Fig 4:**
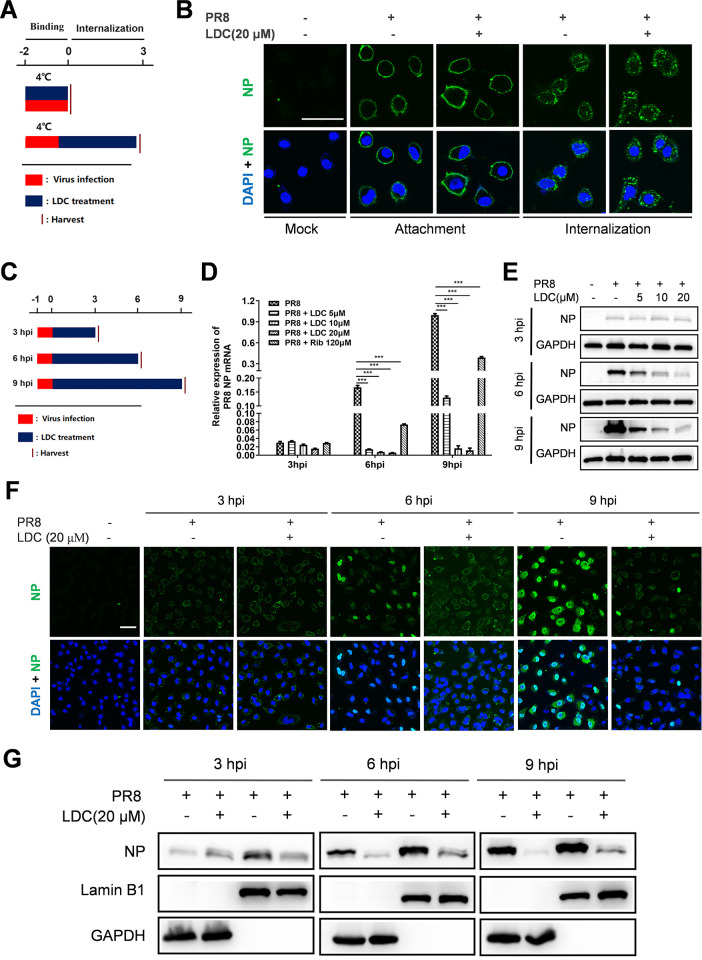
Effects of LDC on viral binding, internalization, and NP nuclear import and translocation. (**A and B**) The experimental design illustration for virus infection and LDC treatment. A549 cells were infected with PR8 at 3 MOI and then cultured in fresh media containing various concentrations of LDC. (**B**) Viral attachment and internalization were examined under confocal microscopy using IFA with Alexa Fluor 488-conjugated goat anti-mouse secondary antibody (green) that binds to mouse anti-NP antibody. Nuclei were counterstained with DAPI (blue). (**C–G**) At specified time points following infection (**C**), RT-PCR was conducted to quantify NP mRNA (**D**), Western blot analysis was performed to assess NP expression (**E**), and IFA using confocal microscopy was employed to visualize the localization of NP (**F**). Additionally, Western blot analysis was carried out after the separation of cytoplasmic and nuclear fractions to analyze the nuclear import of NP (**G**). Scale bar: 50 µm. Statistical significance is denoted by *P < 0.05, **P < 0.01, and ***P < 0.001.

To explore the mechanism of action of LDC on IFV replication, we conducted experiments to assess the impact of LDC during the early and mid-phases of IFV replication. Our initial focus was to determine whether LDC has an influence on the attachment and internalization of the IFV. A549 cells were exposed to a higher dose of IFV (PR8, 3 MOI) in the presence or absence of LDC at 4°C, allowing virus attachment. For internalization, A549 cells were adsorbed with PR8 (3 MOI) at 4°C, followed by incubation with pre-warmed Dulbecco’s modified Eagle’s medium (DMEM) in the presence or absence of LDC at 37°C to facilitate virus internalization (Fig. 4A). The results obtained from IFA indicated that LDC treatment did not alter virus attachment or internalization (Fig. 4B) as no difference was observed between the LDC treated and untreated groups. These observations suggest that LDC does not impede the early stages of viral replication, including virus attachment and internalization.

To determine the impact of LDC treatment on the various stages of the mid-phase IFV replication, we conducted real-time PCR, Western blot analysis, and IFA to examine the transcription of viral mRNA, the expression and localization of viral NP in infected cells. Our findings, depicted in Fig. 4D, showed that LDC treatment significantly reduces the levels of the NP mRNA. Additionally, our investigation indicated that LDC treatment did not affect NP levels at 3 hpi. However, a significant reduction in NP expression was observed at 6 and 9 hpi with LDC treatment (Fig. 4E). Furthermore, the nuclear import of NP determined by IFA was inhibited by LDC treatment, as shown in Fig. 4F. This was further confirmed by the separation analysis of the nuclear and cytoplasmic protein (Fig. 4G). Collectively, our results suggest that LDC inhibits IFV replication in the mid-stage of the viral replication cycle, most likely by interfering with viral RNA transcription and nuclear import of vRNPs.

### LDC downregulates cellular CDK9 expression and suppresses IFV replication in A549 cells

**Fig 5:**
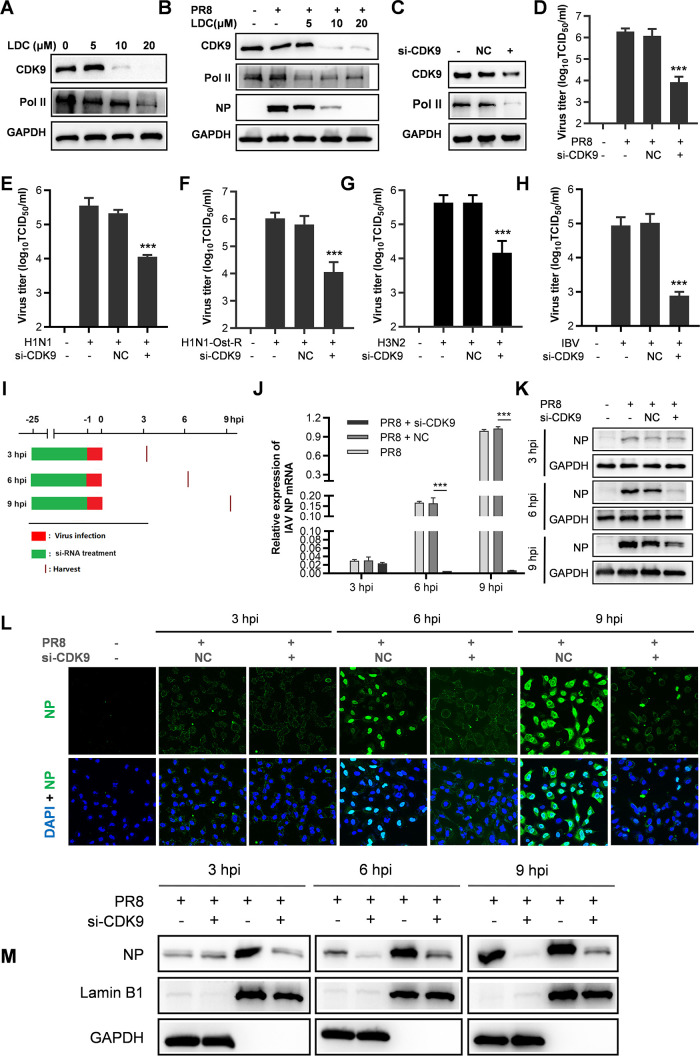
Silencing cellular CDK9 expression decreases IFV replication in A549 cells. (**A and B**) A549 cells cultured in 12-well plates were mock infected (**A**) or infected (**B**) with PR8 at 0.03 MOI for 1 h at 37°C, followed by incubation in fresh media containing LDC at concentrations of 5, 10, or 20 µM. The samples were collected at 24 hpi and subjected to Western blot analysis for CDK9, NP, Pol II, and glyceraldehyde-3-phosphate dehydrogenase (GAPDH) expression levels. (**C**) A549 cells were transfected with siRNA targeting CDK9 (si-CDK9) or non-targeting control siRNA (si-NC) for 24 h, and total protein samples were extracted for CDK9 and protein analysis using Western blot. (D–H) A549 cells were transfected with si-CDK9 or si-NC and then infected with different strains of IFV at 0.03 MOI for 1 h at 37°C, including PR8 (**D**), H1N1 pan2009 (**E**), H1N1 Ost-R (**F**), H3N2 (**G**), and IBV (**H**). The cells were then cultured in fresh media. At 24 hpi, the samples were subjected to viral titer analysis. (**I–M**) A549 cells were transfected with siCDK9 or siNC, followed by PR8 infection at 3 MOI. At indicated time points post-infection (**I**), the samples were collected and subjected to RT-PCR (**J**), Western blot analysis (**K**), IFA using confocal microscopy (**L**), and Western blot analysis after separation of cytoplasmic and nuclear fractions (**M**). Scale bar: 50 µm. Statistical significance is denoted as *P < 0.05, **P < 0.01, and ***P < 0.001.

LDC, a highly specific CDK9 inhibitor, inhibits CDK9-mediated transcription through enhanced pausing of Pol II on genes at the molecular and cellular level (40). We first determined the CDK9 and Pol II protein level in LDC-treated A549 cells and confirmed decreased CDK9 and Pol II expression in both PR8-infected and uninfected cells, as shown in Fig. 5A and B. To exclude the possible off-target effect of LDC and confirm that diminished CDK9 expression could decrease IFV replication, CDK9 siRNAs were used to silence CDK9 expression (Fig. 5C), which clearly led to a decrease in IFV replication, as evidenced by a notable decrease in virus titers against five different IFV strain infections (Fig. 5D through H). Our results support the notion that the reduction of cellular CDK9, achieved through LDC treatment or siRNA silencing, hinders IFV replication in A549 cells.

To investigate the effects of CDK9 silencing on various steps of the mid-phase IFV replication cycle, we assessed the transcription of IFV NP mRNA and the expression of NP protein through RT-PCR and Western blot analysis in CDK9-silenced and IFV-infected cells at 3, 6, and 9 hpi, compared with untreated cells. Similar to the effects observed with LDC treatment on NP expression, we found that siRNA-mediated CDK9 silencing did not influence NP expression at 3 hpi. However, at 6 and 9 hpi, a noteworthy decreased NP expression was evident in cells transfected with siRNA targeting CDK9 (Fig. 5J and K). Additionally, si-CDK9 treatment notably impeded NP’s nuclear import (Fig. 5L). The results presented in Fig. 5M indicate that si-CDK9 significantly inhibits the nuclear import of the primary infected influenza virus. The efficient separation of cytoplasmic and nuclear fractions was confirmed through Western blot analysis using cytoplasmic glyceraldehyde-3-phosphate dehydrogenase (GAPDH) and nuclear (Lamin B1) markers. These outcomes suggest that the downregulation of CDK9 expression affects the mid-phase, including NP’s nuclear import, viral gene transcription, and downstream events of the IFV life cycle.

### LDC treatment protects mice infected with lethal IFV

**Fig 6:**
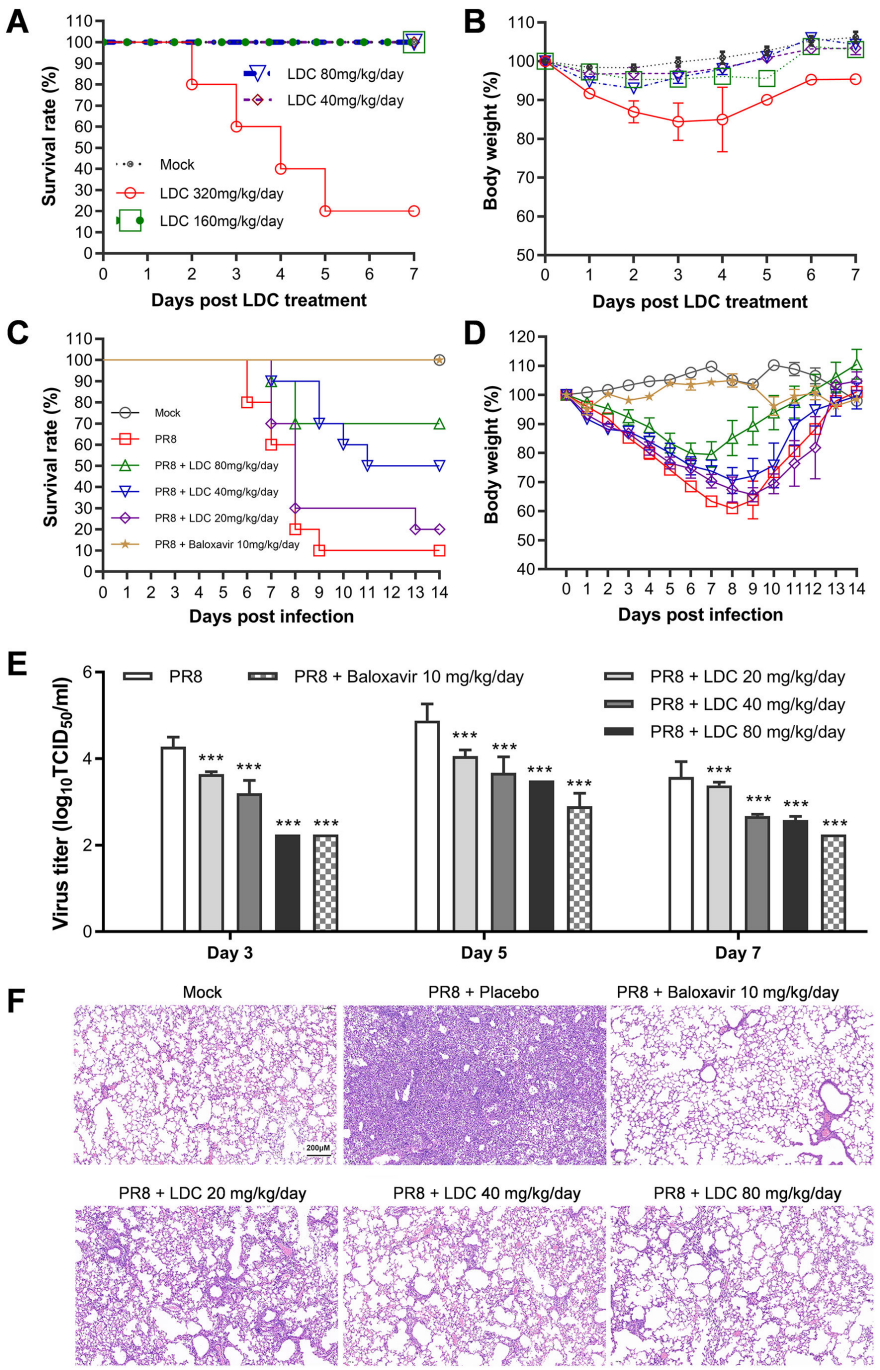
LDC treatment confers protection against lethal PR8 infection in BALB/c mice. (**A and, B**) Toxicological evaluation of LDC *in vivo* with female BALB/c mice. Fifty female BALB/c mice were randomly divided into five groups, and 10 animals in each group received an i.p. injection of LDC at 0, 40, 80, 160, or 320 mg/kg/day. LDC was dissolved in a complex vehicle solution (10% DMSO + 40% PEG300 + 5% Tween 80 + 45% normal saline). Mean survival (**A**) and body weights (**B**) of BALB/c mice after continuous intraperitoneal injection treatment for 4 days. (**C–F**) The mice were infected with 2 LD_50_ of PR8 and subsequently treated twice daily with baloxavir marboxil (10 mg/kg/day, i.g.) or LDC (80, 40, and 20 mg/kg/day, i.p.), starting 4 h prior to infection, for two consecutive days. For the mock infection, 10 mice received treatment with the above complex vehicle solution for two consecutive days. The survival and body weight were monitored daily for 14 days post-infection. Additionally, on days 3, 5, and 7 post-infection, three mice were sacrificed to collect lung samples for virus titer titration using TCID_50_ assay in MDCK cells (*n* = 3). After sacrificing another parallel three mice, hematoxylin and eosin (H&E) staining was used to assess the lung pathology on day 7 post-infection. Statistical significance was determined using the log-rank test (for survival) or Student’s *t*-test (for virus titer) (*P < 0.05; **P < 0.01; ***P < 0.001 compared to the control group treated with the complex vehicle solution.

Before evaluating the therapeutic efficacy of LDC in a mouse model, we measured the toxicity of LDC and observed that all mice survived without any apparent adverse symptoms, such as weight loss, hunching, or fur changes, during 7 days after intraperitoneal injection of a maximum dose of 160 mg/kg/day for four consecutive days (Fig. 6A and B). This high survival rate provides reassurance about the safety of LDC. Next, we evaluated the protection of LDC treatment for two consecutive days on PR8-infected mice. As shown in Fig. 6C, in untreated mice infected with PR8, up to 90% mortality was observed at 14 dpi. However, the mortalities decreased to 80%, 50%, and 30% with LDC treatment at doses of 20, 40, and 80 mg/kg/day, respectively, indicating a significant protective effect of LDC treatment against IFV infection *in vivo*. Baloxavir, used as the positive control in this trial, with its treatment at 10 mg/kg/day, exhibited 100% protection as all mice survived.

The virus titers in the lungs of the infected mice were determined on days 3, 5, and 7 post-IFV infection. The results showed that LDC treatment reduced the virus titer in the lungs of PR8-infected mice in a dose-dependent manner at three monitored time points. Of LDC treatment, 80 mg/kg/day reduced the lung virus titers by 2.0, 1.4, and 1.0 log_10_ at days 3, 5, and 7 post-IFV infection, respectively, compared with respective phosphate-buffered saline (PBS) control. Histopathological assessment of the lungs of virus-infected mice through hematoxylin and eosin (H&E) staining showed that the pulmonary parenchyma and interstitial inflammatory infiltrations in the LDC-treated groups were alleviated compared with the PBS group, with significant improvement in the high-dose group.

The comprehensive results from our *in vivo* efficacy study underscore the protective effects of LDC treatment on mice infected with a lethal dose of IFV. This protection is achieved through inhibiting virus amplification *in vivo* and ameliorating lung damage caused by viral infections.

### The LDC treatment reduces the antibody response in mice to influenza virus infection

B lymphocytes are adaptive immune cells that play a crucial role in the humoral response by producing both non-neutralizing and specific neutralizing antibodies. These antibodies are essential for the clearance of IFV infections through various mechanisms, including neutralization, opsonization, and the activation of antibody-dependent cellular cytotoxicity (41). LDC specifically targets CDK9, which is involved in gene transcription by RNA polymerase II. Inhibiting CDK9 activity may reduce the transcription of genes necessary for B-cell activation and antibody production.

**Fig 7:**
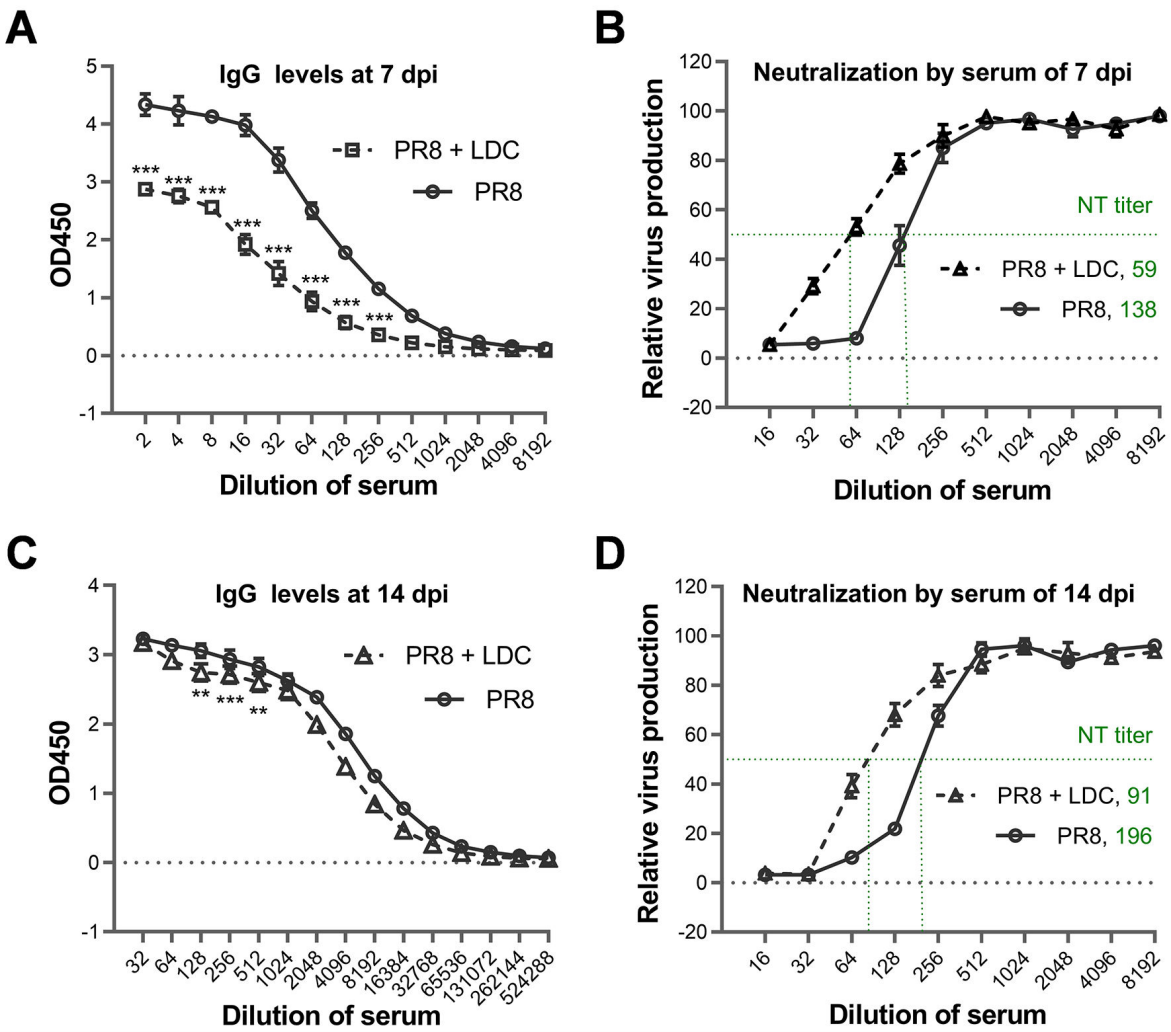
Treatment with LDC led to a decrease in PR8-specific IgG and neutralizing antibody levels. Two groups of BALB/c mice (*n* = 15 per group) were given 1 LD50 of the PR8 virus. One group was treated with LDC at an 80-mg/kg dosage, administered twice daily for two consecutive days starting from day 0 post-infection. The other group did not receive any treatment. Sera were collected from three mice in each group at 7 and 14 dpi, respectively. Influenza virus-specific IgG levels in the collected sera at these time points were determined using enzyme-linked immunosorbent assay (ELISA) by analyzing serial dilutions of each serum sample (**A and C**). Neutralizing antibody titers in the collected sera at 7 and 14 dpi from both the untreated and LDC-treated mice were determined by neutralization assay through analysis of serial dilutions of each serum sample (**B and D**). Statistical significance was assessed using two-way ANOVA (*P < 0.05; **P < 0.01; ***P < 0.001). NT, Neutralization titer.

To investigate the effect of LDC on the antibody response to IFV infection, two groups of BALB/c mice (15 per group) were inoculated with 1× LD_50_ of the PR8 virus. One group received treatment with 80 mg/kg of LDC starting from 0 day post-infection (dpi), administered twice daily for two consecutive days, while another group remained untreated. Sera were collected from three mice in each group at 7 and 14 dpi. The results, depicted in Fig. 7, indicate that LDC-treated mice exhibited significantly suppressed serum IgG production at 7 dpi (Fig. 7A) and slight suppression at 14 dpi (Fig. 7C) compared to the untreated mice. Therefore, the suppression of antibody production by LDC seems to diminish after the cessation of treatment. Analysis of neutralization antibody production, as determined by neutralization assay over a serially diluted mouse serum, revealed a twofold difference in neutralization titer between the LDC-treated and untreated groups at 7 dpi (Fig. 7B) and 14 dpi (Fig. 7D), with neutralization titers of 59 and 138 at 7 dpi and 91 and 196 at 14 dpi, respectively. Hence, mice subjected to LDC treatment exhibit a prolonged decrease in neutralization levels.

## DISCUSSION

IFV infection poses a significant threat to human health, with seasonal epidemics and occasional global pandemics. While current vaccines and antiviral drugs are available, they are only partially effective due to the rapid changes in viral epitopes and the rise of drug-resistant strains (4, 42). Therefore, there is an urgent need to explore new strategies to combat IFV infections. Here, we first reported that LDC, a highly specific CDK9 inhibitor, potently inhibits the replication of five different IFV strains in A549 cells, including oseltamivir-resistant PR8 and influenza B virus strains, with EC_50_ ranging from 3.92 to 6.31 µM (Fig. 1 and 2; Table 2). CDK9 knock-down resulted in a significant reduction in IFV replication in A549 cells (Fig. 5), suggesting the antiviral effect of LDC is an on-target effect. Importantly, LDC treatment protected mice from lethal PR8 infection, with a 60% increase in mouse survival. It also dramatically suppressed viral replication in PR8-infected mouse lungs (Fig. 6). Mechanistically, we revealed that LDC treatment and CDK9 silencing suppress IFV replication by disrupting the nuclear import of vRNPs and mRNA transcription (Fig. 4 and 5). Altogether, our findings indicate that CDK9 could be a promising target for the development of anti-IFV drugs, and LDC is a potential anti-IFV candidate.

**TABLE 2:** Real-time PCR primer sequences

Name^*a*^	Sequence
NP-F	5′-AGCATTGTTCCAACTCCTTT-3′
NP-R	5′-GACGATGCAACGGCTGGTCTG-3′
18S rRNA-F	5′-AACGGCTACCACATCCAAGG-3′
18S rRNA-R	5′-GGGAGTGGGTAATTTGCGC-3′

^*a*^ F, forward primer; R, reverse primer.

CDK9 is a known regulator of cellular transcription, and it has been revealed to participate in the replication process of multiple viruses. A study reported by Yamamoto et al. revealed that a CDK9 inhibitor, FIT-039, suppressed replication of a broad spectrum of DNA viruses through inhibition of mRNA transcription, including HSV, human cytomegalovirus, and human adenovirus in cultured cells. Specifically, topical application of FIT-039 alleviated skin lesion in an HSV-1 infection mouse model (43). CDK9 was also shown to be involved in human immunodeficiency virus (HIV) replication via participating in the transactivation of the HIV-Tat protein (44). These studies suggest that CDK9 might facilitate viral replication and serves as a potential antiviral target. However, the function of CDK9 in IFV infection is largely unknown. In the present study, we showed for the first time that CDK9 inhibition by LDC treatment and CDK9 silencing inhibit IFV replication (Fig. 1 and 5). CDK9 is an essential kinase for the transcription of Pol II-dependent genes, and Pol II serves as the source of nascent capped host RNAs targeted by IFV (36, 45). Previous research has validated that two Pol II inhibitors, 5,6-dichloro-1-b-D-ribofuranosyl-benzimidazole and alpha-amanitin, inhibit IFV replication in chick embryo fibroblast cells by interfering with viral RNA transcription (46, 47). Here, our study revealed that inhibition of CDK9 through LDC treatment or CDK9 silencing leads to a reduction in RNA pol II expression (Fig. 5), subsequently resulting in the inhibition of viral NP mRNA transcription (Fig. 4E and 5J).

Moreover, our study showed that the nuclear import of vRNPs is hindered by LDC treatment and CDK9 silencing (Fig. 4F, G, 5L and M), indicating that the absence or dysfunction of CDK9 impairs the nuclear import of vRNPs. IFV enters host cells through receptor-mediated endocytosis and then transports into the cell nucleus via the cellular importin-α/β nuclear import pathway. Nuclear import of vRNPs is critical for IAV infection because viral RNA replication occurs in the nuclei. Previous research has identified that several host proteins, including BinCARD (40), ABTB1 (25), MOV10 (48), PLSCR1 (49), and eEF1D (50), are involved in regulating the transport of vRNPs or newly synthesized NP into the nucleus of infected cells. Our present study identified CDK9 acting as a new host factor involved in the nuclear import of vRNPs, although the underlying mechanism remains to be explored. Collectively, our findings indicate that IFV hijacks CDK9 for the benefit of its replication via promoting the nuclear import of vRNPs and viral RNA transcription, and CDK9 might be a promising anti-IFV target.

Although no CDK inhibitors have yet been approved for the treatment of viral infections, there are CDK inhibitors that have been approved as anticancer drugs in recent years. Currently, three CDK4/6 inhibitors, palbociclib, ribociclib, and abemaciclib, have been approved as drugs for treating breast cancer by the U.S. Food and Drug Administration. Palbociclib, in particular, has emerged as a blockbuster first-line drug in breast cancer therapy, with the global market surpassing US$5 billion in 2020 (51). In our animal trial of evaluating acute toxicity of LDC in mice, consecutive intraperitoneal injection of 160 mg/kg of LDC for 4 days did not lead to any apparent adverse effects in mice, except for a temporary and minor body weight loss. Notably, a 2-day intraperitoneal injection of 80 mg/kg of LDC effectively protected mice infected with lethal influenza. It was evident that a 2-day treatment of LDC was significantly less toxic than a 4-day treatment. These findings indicate a satisfactory safety profile of LDC *in vivo*. Moreover, if the LDC treatment is administered through nasal inhalation, the toxic side effects can be further reduced compared to systemic administration, potentially enhancing the safety and efficacy of LDC treatment. In addition to the antiviral research, we assessed the potential effects of LDC treatment on the virus-specific antibody levels and the neutralization antibody titers. Our results suggested that LDC treatment leads to a significant reduction in antibody production in PR8-infected mice during the early phase of antibody response, which is likely associated with the reduced viral titers in the lungs of LDC-treated PR8-infected mice. However, it is important to note that the diminished antibody response may also be linked to the direct modulation of cytokine production and immune response signaling pathways by LDC treatment, potentially impairing the immune response and subsequent antibody production. Further research is required to validate this hypothesis. Moreover, the influence of treatment with LDC or other CDK inhibitors, known for their antiviral activity against influenza infections, on the body’s immunity to reinfection by influenza remains to be investigated.

In conclusion, our study shows that CDK9 inhibitor LDC potently suppresses IFV replication *in vitro*, including H1N1 Ost-resistance and IBV strains, and protects mice from lethal IFV infection. In parallel, we have elucidated the underlying mechanisms of LDC against IFV infection as interfering viral RNA transcription and nuclear import of vRNPs. This indicates that LDC possesses a broad-spectrum anti-influenza characteristic with a minimum tendency to induce resistance. Our findings suggest that CDK9 might be a promising antiviral target, and LDC presents excellent potential as a novel anti-influenza candidate.

## MATERIALS AND METHODS

### Compounds and reagents

The compound LDC000067 (LDC) was acquired from Shanghai Bide Pharmatech Co., Ltd., with a purity of 99.3% (Shanghai, China). For *in vitro* experiments, LDC was solubilized in 100% dimethyl sulfoxide (DMSO) and then diluted with culture medium to ensure that the final concentration of DMSO did not exceed 0.4%. For *in vivo* experiments, LDC was dissolved in a solution containing 10% DMSO, 40% PEG300, 5% Tween-80, and 45% saline. Baloxavir marboxil was procured from Selleck Chemicals Co., Ltd. (Shanghai, China), while ribavirin (Rib) was obtained from Star Lake Bioscience Co., Ltd. (Zhaoqing, China).

### Cells and viruses

Human lung epithelial (A549) cells and Madin–Darby canine kidney (MDCK) cells were purchased from the Center of Cellular Resource, Chinese Academy of Sciences in Shanghai, China. The cells were sustained in DMEM (Gibco, USA), supplemented with 1% penicillin/streptomycin (Beyotime, Shanghai, China) and 10% fetal bovine serum (MeilunBio, Shanghai, China) at 37°C with 5% CO_2_. Influenza A virus subtypes, namely, avian strains A/Puerto Rico/8/1934 (PR8), oseltamivir-resistant A/PuertoRico/8/1934 (H274Y) (H1N1-Ost-R), A/human/Hubei/1/2009 (H1N1 pan2009), A/human/Hubei/3/2005 (H3N2), and influenza B virus subtypes B/human/Hubei/1/2007 (IBV) were included in the study. The stock virus was cultivated in 10-day-old embryonated chicken eggs for 48 h at 37°C. Subsequently, the allantoic fluid was retrieved, and aliquots were stored at −80°C until required (52, 53). Subsequent titration of the viruses in MDCK cells facilitated the determination of the 50% tissue culture infectious dose (TCID_50_) using the Reed and Muench method (54).

### Mice

We acquired female BALB/C mice (6 to 8 weeks old) from the Guangdong Medical Laboratory Animal Center (GDMLAC, Guangzhou, China). These mice were housed in pathogen-free isolators, and the Institutional Animal Care and Use Committee at Jinan University approved the housing conditions.

### Animal experiments

For a toxicological evaluation of LDC *in vivo*, female BALB/c mice (6 weeks old) were injected intraperitoneally with LDC at the specified dose for four consecutive days. Throughout the treatment period, all mice were monitored daily for survival and weight changes for a duration of 7 days.

For the assessment of *in vivo* therapeutic efficacy, female BALB/c mice (6 weeks old) were infected with 2 LD50 of the mouse-adapted influenza virus A/Puerto Rico/8/1934 (H1N1) and subsequently received drug treatment twice daily at the specified doses. Treatment commenced 4 h prior to infection and continued for two consecutive days. The mice’s survival and body weight were monitored daily for 14 days post-infection. Additionally, three mice were euthanized on days 3, 5, and 7 post-infection to collect bronchoalveolar lavage fluids for virus titer titration. Furthermore, lung pathology was assessed using H&E staining in another three mice sacrificed on day 7 post-infection.

### Antibodies

Influenza A nucleoprotein antibody (NP protein) was purchased from Sino Biological Inc. (Beijing, China). The CDK9 antibody and goat anti-mouse conjugated with Alexa Fluor488 (green) were acquired from Cell Signaling Technology, Inc. (MA, USA). Additionally, the Pol II, horseradish peroxidase (HRP)-labeled goat anti-mouse IgG (H + L), and HRP-labeled goat anti-rabbit IgG (H + L) were obtained from Beyotime Biotechnology in Shanghai, China. Finally, the GAPDH mouse monoclonal antibody was sourced from Proteintech Group, Inc. (Wuhan, Hubei, China).

### Cytotoxicity and antiviral assays

MDCK or A549 cells (2.5 × 10^5^) were cultured in 96-well plates until reaching full confluency. Subsequently, the medium was replaced with fresh medium containing serially diluted LDC compounds, and the cells were then further incubated for 48 h. The Cell Titer-Glo cell viability assay kit (Promega, Beijing, China) was employed to assess compound toxicity. The Cell Titer-Glo substrate was reconstituted in the Cell Titer-Glo buffer, equilibrated to room temperature, and then added to the cell cultures, followed by an incubation at 37°C for 10 min. The absorbance was then measured at 450 nm using a microplate reader (Thermo Fisher Scientific, MA, USA). Three independent experiments were conducted in duplicate to determine the 50% cell cytotoxic concentration (CC_50_), and the data were analyzed using Prism v.6 software.

MDCK and A549 cells (2.5 × 10^5^) were infected with IFV (PR8) at 37°C for 1 h and treated with LDC. Following a 24-h incubation period, viral titers were assessed using the 50% tissue culture infectious dose (TCID_50_) method in MDCK cells. Additionally, the morphological changes in virus-infected cells were examined using microscopy.

### Viral titer titration assay

The viral titer was determined using the endpoint dilution assay as previously described (55). In this assay, viral preparations were diluted in 10-fold increments in essential media. Subsequently, 100 µL of each diluted preparation was introduced in quadruplicate to confluent monolayers of MDCK cells in 96-well plates, followed by a 2-h incubation at 37°C. After aspirating the inoculum from each well, the cell monolayers were replenished with fresh essential media and cultured for 72 h. The endpoint was determined based on NA activity, and the virus titer was then calculated and expressed as a 50% tissue culture infective dose (TCID_50_/mL).

### Indirect immunofluorescence assay (IFA)

For immunostaining, the PR8-infected or uninfected cells were fixed with 4% paraformaldehyde for 10 min, permeabilized with 0.25% Triton X-100 for 10 min at room temperature (RT), blocked with 1% bovine serum albumin (BSA) for 1 h at RT, and then incubated with a mouse monoclonal antibody against the NP of IFV (IgG2b Clone, 1:500 dilution, Sino Biological, Beijing, China) at 4°C overnight. After three washes with PBS, the cells were incubated for 1 h at RT with goat anti-mouse conjugated with Alexa Fluor488 (green) (Cell Signaling Technology, MA, USA) at 1:1,000 dilution. Nuclei were counterstained using 50 µL of 4,6-diamidino-2-phenylindole (DAPI, 300 nM; Sigma-Aldrich, Taufkirchen Germany) (blue). Immunofluorescence was captured using a Leica DMI 4000B fluorescence microscope or TCS SP8 confocal laser scanning microscope (Leica, Wetzlar, Germany) (56).

### Real-time quantitative reverse-transcription PCR (qRT-PCR)

Total RNA extraction was done using the Total RNA Rapid Extraction Kit (Fastagen, Shanghai, China) following the manufacturer’s instructions. Subsequently, the first strand of cDNA was synthesized using Super Script III Reverse Transcriptase (Genstar, Beijing, China) and reverse transcribed with primers specified in Table 2. PCR amplification was conducted on 1 µL of reverse-transcribed product using primers designed for IFV-NP, IFV-M2, and 18S rRNA, as outlined in Table 2 (57). Real-time PCR was performed utilizing Real Star Green Power Mixtures containing SYBR Green I Dye (Genstar, Beijing, China) on the CFX96 Real-time PCR system (Bio-Rad, CA, USA). Relative mRNA expression was determined using the 2^−ΔΔCT^ method, with DMSO-treated infected cells or DMSO-treated mock-infected cells as reference samples for ascertaining IFV-NP and 18S rRNA gene expression, respectively.

### Western blot analysis

Upon treatment, whole-cell lysates were subjected to lysis in RIPA lysis buffer containing 1 mM phenylmethylsulfonylfluoride at 4°C. Nuclear or cytoplasmic extracts were prepared using Nuclear and Cytoplasmic Protein Extraction Kit (Beyotime, Shanghai, China) following the manufacturer’s protocol. Subsequently, the supernatant was obtained through centrifugation at 13,000 rpm for 15 min at 4°C, and the total protein content was quantified using the BCA protein assay kit (Beyotime, Shanghai, China). After this, 10 μg of total protein from each sample underwent electrophoresis on a 10% SDS-PAGE gel and were transferred to polyvinylidene-fluoride membranes. Post-transfer, the membranes were subjected to blocking and subsequently incubated with the specific antibodies at specified dilutions and conditions: IFV NP Mouse Monoclonal Antibody (IgG2b Clone, Sino Biological, Beijing), CDK9 Rabbit Monoclonal Antibody (Cell Signaling Technology, MA, USA), Pol II Mouse Monoclonal Antibody (Beyotime, Shanghai, China), Lamin B1 Rabbit Monoclonal Antibody (Beyotime, Shanghai, China), and GAPDH Mouse Monoclonal Antibody (Proteintech, Wuhan, China) at 4°C overnight. Subsequently, HRP-conjugated goat anti-mouse or anti-rabbit IgG (H-L) (Beyotime, Shanghai, China) was utilized as the secondary antibody during a 1-h incubation at room temperature. The visualization of Western blot results was achieved using chemiluminescence technology.

### IFV attachment and internalization assay

A549 cells were pre-chilled at 4°C for 1 h, following which the medium was replaced with DMEM containing IFV (PR8, 3 MOI) with or without LDC at the indicated concentrations. Subsequently, the cells were incubated for another 2 h at 4°C to facilitate virus attachment. Three washes with PBS succeeded in eliminating any unbound virus particles and chemicals. Finally, the cells underwent analysis for viral NP using laser scanning confocal microscopy.

A549 cells were pre-chilled at 4°C for 1 h and subsequently incubated in DMEM containing IFV (PR8, 3 MOI) at 4°C for 2 h, a time frame conducive to virus binding but not virus internalization. Following three washes with PBS, the cells were transferred to fresh medium with or without LDC and transitioned to 37°C for an additional 3-h incubation to promote virus internalization. Subsequently, the cells underwent analysis for viral NP using laser scanning confocal microscopy.

### Gene silencing with siRNA

GenePharma Biotech (Shanghai, China) synthesized the siRNAs targeting CDK9 (si-CDK9) and the control siRNA (siNC) used in this study. The siRNA sequences are as follows: si-CDK9, 5′-CCCUCAACCACGACUUCUUTT-3′; si-CDK9 antisense, 5'-AAGAAGUCGUGGUUGAGGGTT-3'; the nontargeting control siRNA (siNC), 5′-UUCUCCGAACGUGUCACGUTT-3′. A549 cells were transfected with 50 nM siRNA utilizing 5 µL of Lipofectamine 3000 Transfection Reagent (Thermo Scientific, CA, USA).

### Quantification of influenza virus-specific IgG levels in mouse sera

The PR8 virus was purified and inactivated using β-Propiolactone (CAS: 57–57-8, MCE) at a ratio of 1:3,000 (vol/vol). The inactivated PR8 virus (2 µg per well) was immobilized onto a 96-well enzyme-linked immunosorbent assay (ELISA) plate (Corning, USA) and incubated overnight at 4°C. Subsequently, the plate was subjected to five washes and then blocked with 1% BSA for 1 h at 37°C. After five additional washes, the plate was incubated with 100 µL of serially diluted sera at 37°C for 2 hours. The plate then underwent five more washes before being incubated with 100 µL of 1,000-fold diluted horseradish peroxidase (HRP)-labeled Goat Anti-Mouse IgG(H + L) (Beyotime, Shanghai, China) at 37°C for 1 h. Subsequently, after five washes, 100 µL of TMB (3,3′,5,5′-tetramethylbenzidine) substrate (Beyotime, Shanghai, China) was added to each well until the solutions turned blue, at which point 100 µL of Stop Solution for TMB Substrate (Sulfuric acid free) (Beyotime, Shanghai, China) was added to halt the reaction. Finally, the absorbance was measured at OD450 nm using the Varioskan LUX multimode microplate reader (Thermo Scientific, Waltham, MA, USA) (47).

### Neutralization assay

The neutralization assay was performed to measure the neutralizing antibody levels of sera from mice infected with the influenza virus. The process involved diluting the sera and heat-inactivating them at 56°C for 30 min. The inactivated sera were mixed with 100 TCID_50_ of the PR8 virus and incubated at 37°C for 1 h. After that, the mixture was added to MDCK cells that had been pre-seeded and left to incubate at 37°C for an additional hour. Following this incubation, the medium was replaced with fresh DMEM, and the cells were further cultured at 37°C for 24 h. The amount of virus produced in the supernatant was determined by measuring the NA activity.

### Statistical analysis

All experiments were conducted a minimum of three times, and the results were reported as mean ± standard deviation (SD). Statistical significance was assessed using Student’s *t*-test for comparing two groups and the one-way analysis of variance (ANOVA) for comparing more than two groups. Statistical significance was denoted by *P < 0.05, **P < 0.01, and ***P < 0.001. These levels of significance were considered statistically significant.
